# Comparison of Food-Based and Music-Based Regulatory Strategies for (Un)Healthy Eating, Depression, Anxiety and Stress

**DOI:** 10.3390/nu14010187

**Published:** 2021-12-31

**Authors:** Kamila Czepczor-Bernat, Adriana Modrzejewska, Justyna Modrzejewska, Rafał Majzner

**Affiliations:** 1 Institute of Psychology, University of Wrocław, 50-527 Wroclaw, Poland; 2 Department of Psychology, School of Health Sciences in Katowice, Medical University of Silesia in Katowice, 40-055 Katowice, Poland; adriana.modrzejewska@sum.edu.pl; 3 Institute of Pedagogy, University of Bielsko-Biala, 43-309 Bielsko-Biala, Poland; jmodrzejewska@ath.bielsko.pl (J.M.); rmajzner@ath.bielsko.pl (R.M.)

**Keywords:** depression, anxiety, stress, (un)healthy eating, music, eating, food-based and music-based emotional regulation strategy

## Abstract

There are many ways to regulate emotions. People use both adaptive (e.g., regulation by music) and maladaptive (e.g., regulation by food) strategies to do this. We hypothesized that participants with a high level of food-based regulatory strategies and a low level of music-based regulatory strategies (a group with the least adaptive form of emotion regulation) would have significantly greater levels of unhealthy eating behaviours, depression, anxiety and stress, as well as a significantly lower level of healthy eating behaviours than those with a low level of food-based regulatory strategies and a high level of music-based regulatory strategies (a group with the greatest adaptive form of emotion regulation). Participants (*N* = 410; *M*_age_ = 31.77, *SD* = 13.53) completed: the Brief Music in Mood Regulation Scale, the Emotional Overeating Questionnaire, the Healthy and Unhealthy Eating Behavior Scale, the Depression, Anxiety and Stress Scale and a socio-demographic survey. The four clusters were identified: (a) Cluster 1 (*N* = 148): low food-based regulatory strategies and high music-based regulatory strategies; (b) Cluster 2 (*N* = 42): high food-based regulatory strategies and high music-based regulatory strategies; (c) Cluster 3 (*N* = 70): high food-based regulatory strategies and low music-based regulatory strategies; (d) Cluster 4 (*N* = 150): low food-based regulatory strategies and low music-based regulatory strategies. Overall, our outcomes partially support our hypothesis, as higher levels of unhealthy eating behaviours, depression, anxiety and stress were observed in participants with high food-based and low music-based regulatory strategies as compared with adults with low food-based and high music-based regulatory strategies. To sum up, the results obtained indicate that during the COVID-19 pandemic the group of people regulating their emotional state and unhealthy eating predominantly with food is potentially characterized by worse functioning than the group of people regulating with music. Therefore, it can be concluded that people who regulate their functioning using food should be included in preventive measures by specialists. During the visit, psychologists and primary care physicians can ask patients about their daily strategies and based on this information specialists can estimate the potential risk of developing high levels of stress and anxiety, depressive disorders and unhealthy eating habits and provide specific (match) intervention.

## 1. Introduction

There are many ways to regulate emotions [1]. People use both adaptive (e.g., regulation by music-listening to music) and maladaptive (e.g., regulation by unhealthy food or/with uncontrolled eating, excessive in intensity, frequency of eating) strategies to do this [1,2,3,4]. Some of these strategies can have serious consequences [4]. For example, the long-term food-based emotional regulation strategy may be related to (over)eating unhealthy foods (e.g., comfort food incl. sweets, fast food etc.) [3,5,6], which in turn contributes to the development of obesity [7,8] and as a consequence significantly increases the risk of many somatic diseases [9]. Therefore, strategies should be sought that will have a more positive effect on health and will benefit both short-term and long-term physical and mental functioning [4].

One of the adaptive ways may be a music-based emotional regulation strategy [10,11]. Previous reports indicate that this strategy (listening to music) may be effective in changing one’s emotional state [10,12,13,14,15]. Listening to music can affect one’s emotional state through a number of neurotransmitter changes that are associated with reducing stress, achieving a state of stress relief and improving mood [11,12,13]. Interestingly, some authors indicate that these changes are similar to the situation in which food-based strategies are used [13,16,17,18].

Analysing information relating to the regulation of emotions can be especially relevant during the pandemic [10,19]. This is because numerous studies show that the methods of regulating emotions used so far (e.g., face-to-face meetings with friends, regular physical activity) are often limited by restrictions related to COVID-19 [20,21,22]. At the same time, other studies show that people around the world declare that COVID-19 is a source of severe stress and anxiety for them [23,24,25]. Moreover, it also turns out that the pandemic contributed to an increase in the intensity of depressive symptoms [24,25,26,27]. In this context, it should be mentioned that there are studies that show that listening to music can be an effective mood regulator during the COVID-19 period [19]. Moreover, although numerous studies show that the effectiveness of music therapy in the treatment of various mental disorders is confirmed, there is still a lack of knowledge about the results of the independent use of music (“self-therapy”—activity not therapist-guided) [2,11].

It turns out that people from all over the world use music as a regulator of emotional states [19]. Interestingly, some reports indicate that the ability to employ adaptive regulation of emotions can effectively promote healthy eating habits [4,28]. Moreover, there are also hypotheses that listening to music can be a much healthier alternative to emotional eating [29]. However, there is a lack of research directly comparing the emotional functioning of people using these strategies (food-based strategy vs. music-based strategy) and research checking whether both of these strategies differentiate healthy and unhealthy eating behaviours. Therefore, this study aimed to: (1) classify different conditions associated with food-based (emotional overeating) and music-based (listening to music) emotional regulation strategy and (2) evaluate and compare the severity of (un)healthy eating and emotional state (depression, anxiety, stress) in individuals with the different emotional regulation strategies mentioned above. We hypothesised that participants with a high level of food-based regulatory strategies and a low level of music-based regulatory strategies (a group with the least adaptive form of emotion regulation) would have significantly greater levels of unhealthy eating behaviours, depression, anxiety and stress, as well as a significantly lower level of healthy eating behaviours than those with a low level of food-based regulatory strategies and a high level of music-based regulatory strategies (a group with the greatest adaptive form of emotion regulation).

## 2. Materials and Methods

### 2.1. Participants and Procedure

This study was performed in line with the principles of the Declaration of Helsinki. Approval was granted by the Ethics Committee (no. 2021/5/5E/6). Recruitment was conducted online among volunteers via an online advertisement on social media from May to June 2021. After contacting the researchers, participants were made aware that participation in the study was anonymous and voluntary and were informed about the aims of the study. Subsequently, if they maintained their willingness to participate in this study, they were asked to approve the declaration of informed consent. Those who approved it were sent a link to the online survey (populated on Google Forms).

Four hundred and ten adults participated in the study ranging in age from 18 to 77 years (M = 31.77, SD = 13.53) and in body mass index (BMI) from 15.02 to 46.51 kg/m^2^ (M = 24.03, SD = 4.99). In relation to BMI, participants were: (a) underweight: 31 (7.56%), (b) normal weight: 236 (57.56), (c) overweight: 93 (22.68%), (d) obese: 50 (12.20%). In terms of education, participants had: (a) elementary education: 1 (0.24%), (b) vocational education: 4 (0.98%), (c) secondary education: 160 (39.02%), (d) higher education: 245 (59.76%).

### 2.2. Measures

Five measures were included in the online survey. Permission to use all questionnaires has been obtained from the authors. To get a Polish language version for questionnaires that did not have one, we used the standardized back-translation procedure [30]. Validity was assessed for all these measures by a confirmatory factorial analysis (CFA) using AMOS (SPSS). For all questionnaire, in CFA results, the model fit indices are acceptable (RMSEA < 0.05; CFI, GFI, TLI > 0.95).

#### 2.2.1. The Brief Music Regulation Scale

To measure music-based emotional regulation strategy, participants completed the Brief Music in Mood Regulation Scale (B-MMRS) [11]. This scale consists of the total score and following 7 subscales: (a) entertainment: happy mood maintenance, (b) revival: relaxation and new energy, (c) strong sensation: intense emotion induction, (d) diversion: distraction from worries and stress, (e) discharge: release and venting of negative emotion, (f) mental work: contemplation and reappraisal of emotional experience, (g) solace: emotional validation and support when feeling down. The higher scores reflected a stronger tendency to regulate emotions by using music. All items were rated on a 5-point scale ranging from 1 (strongly disagree) to 5 (strongly agree) [11]. In this study, we used only the total score for which McDonald’s ω (as the reliability coefficients) was 0.95.

#### 2.2.2. The Emotional Overeating Questionnaire

To measure food-based emotional regulation strategy, participants were asked to complete the Emotional Overeating Questionnaire (EOQ) [31]. The EOQ includes the following 9 states in response to which people overate during the last 28 days: anxiety, loneliness, sadness, anger, tiredness, happiness, guilt, boredom and physical pain. The higher scores reflected a stronger tendency to regulate emotions by using food. All items were rated on a 7-point scale ranging from 0 (no days) to 6 (every day) [31]. McDonald’s ω (as the reliability coefficients) was 0.87.

#### 2.2.3. The Healthy and Unhealthy Eating Behavior Scale

To measure healthy and unhealthy eating behaviours, participants were asked to complete the Healthy and Unhealthy Eating Behavior Scale (HUEBS) [32]. The HUEBS comprises two subscales: (a) healthy eating (HE), (b) unhealthy eating (UE). This scale evaluates the extent to which people generally consume different healthy and unhealthy food items. The higher scores reflected a greater level of healthy and unhealthy eating behaviours. All items were rated on a 7-point scale ranging from 1 (never) to 7 (always) [32]. McDonald’s ω (as the reliability coefficients) for HUEBS-HE scores was 0.86 and for HUEBS-UE 0.87.

#### 2.2.4. The Depression, Anxiety and Stress Scale

To measure depression, anxiety and stress, participants were asked to complete the Depression, Anxiety and Stress Scale (DASS) [33,34]. The DASS comprises three subscales: (a) depression (D), (b) anxiety (A), (c) stress (S). This scale evaluates one’s emotional state over the previous week. The higher scores reflected a greater level of depression, anxiety and stress. All items were rated on a 4-point scale ranging from 0 (did not apply to me at all) to 3 (applied to me very much, or most of the time) [33,34]. McDonald’s ω (as the reliability coefficients) for DASS-D was 0.95, for DASS-A 0.91 and for DASS-S 0.95.

#### 2.2.5. The Demographic Survey

Participants also completed the following information: age, educational qualification, weight and height. The body mass index was calculated based on self-report data. The validity of the use of self-report dates is confirmed, inter alia, by Davies et al. [35], after obtaining results that showed good agreement between the self-report data and direct anthropometric measurements.

### 2.3. Statistical Analysis

We conducted a two-step cluster analysis (with Schwarz’s Bayesian criterion, BIC) [36] using IBM SPSS Statistic version 26. This analysis was used to identify clusters based on food-based and music-based regulatory strategies. Multivariate analysis of variance (MANOVA) with the Bonferroni corrected/adjusted *p*-value was used to assess differences between the clusters with regard to eating behaviours (healthy and unhealthy eating subscales) and emotional state (depression, anxiety and stress subscales). In addition, we also used Cramer’s V as a measure of association (that carry out the adjustment using 𝜒^2^). We applied the most commonly used significance level*: p*-value < 0.05. 

## 3. Results

### 3.1. Cluster Analysis of Food-Based and Music-Based Regulatory Strategies

The four clusters were identified: (a) Cluster 1 (*N* = 148): low food-based regulatory strategies (*M* = 3.25) and high music-based regulatory strategies (*M* = 70.82); (b) Cluster 2 (*N* = 42): high food-based regulatory strategies (*M* = 16.38) and high music-based regulatory strategies (*M* = 70.50); (c) Cluster 3 (*N* = 70): high food-based regulatory strategies (*M* = 22.04) and low music-based regulatory strategies (*M* = 33.77); (d) Cluster 4 (*N* = 150): low food-based regulatory strategies (*M* = 5.22) and low music-based regulatory strategies (*M* = 37.89). Table 1 shows the demographic characteristics of these clusters.

### 3.2. Comparison of the Four Clusters for (Un)Healthy Eating, Depression, Anxiety and Stress

The MANOVA statistical test indicated significant differences among the cluster considered for eating behaviours (healthy and unhealthy eating scales), *V* = 0.10, *F*(2, 405) = 7.02, *p* < 0.001, and emotional state (depression, anxiety and stress scales), *V* = 0.14, *F*(3, 404) = 6.44, *p* < 0.001. The results with Bonferroni’s adjustment for multiple comparisons are presented in Table 2.

Referring to the selected, most important results, with regard to healthy eating behaviours, we did not find significant differences between all clusters. In relation to unhealthy eating behaviours, Cluster 3 had a significantly higher score than Cluster 1 and 4. Moreover, Cluster 1 had a significantly lower level of unhealthy eating behaviours than Cluster 4. In respect of the emotional state, Cluster 3 had a higher level of depression, anxiety and stress than Cluster 1 and 4. In addition, Cluster 1 had significantly lower scores than Cluster 2. Only, in relation to anxiety, Cluster 2 differed significantly only from Cluster 4, and the first of these groups had a higher score for this variable. The other comparisons were insignificant.

Overall, our outcomes partially support our hypothesis, as higher levels of unhealthy eating behaviours, depression, anxiety and stress were observed in participants with high food-based and low music-based regulatory strategies as compared with adults with low food-based and high music-based regulatory strategies.

## 4. Discussion

The key results have been related to the fact that participants with a high level of food-based regulatory strategies and a low level of music-based regulatory strategies (Cluster 3—the group with the least adaptive form of emotion regulation) had significantly greater levels of unhealthy eating behaviours, depression, anxiety and stress than those with a low level of food-based regulatory strategies and a high level of music-based regulatory strategies (Cluster 1—the group with the greatest adaptive form of emotion regulation). It, therefore, shows that the most of findings of this study confirmed our hypothesis. We did not observe significant differences between Clusters 1 and 3 only in relation to healthy eating behaviour.

In our study, we compared people who used two common methods of regulation for eating behaviours and emotional state–food-based (emotional overeating) and music-based (listening to music) strategies. Our results show that during the COVID-19 pandemic, the participants who regulate their functioning in a dominant way with food (Cluster 3) are potentially characterised by worse emotional functioning and more unhealthy eating behaviour than the group who regulate their functioning in a dominant way with music (Cluster 1). This implies that listening to music in certain situations may be more beneficial in response to negative affective states [10] than emotional eating. The lower scores in depression, anxiety and stress in relation to music listening are confirmed by studies on the efficacy of treating depressive syndromes and increasing quality of life in depressed patients [37].

At this point, it should be emphasized that in an increasingly obesogenic environment, searching for factors conducive to making healthy food choices is crucial [28]. What is important is that one factor that contributes to inappropriate eating behaviour are the emotions people experience. Therefore, it can be concluded that coping with emotions is one of the most crucial human adaptive behavioural skills [38], so it is important to analyse how people deal with emotional states and tensions [4,28].

It should be mentioned at this point that on the one hand previous research indicates that maintaining a healthy diet is often compromised by our decisions in emotionally charged situations [39] and in the context of maladaptive emotion regulation food-based emotion regulation is often and widely chosen by people [8,40,41]. Moreover, it is also confirmed that negative emotions (such as loneliness, anger and anxiety) can be reduced in this way [42] and interestingly, depression is also counted among those disorders whose reduction is mediated by eating [42,43]. On the other hand, listening to music is the second, most effective method of regulation of bad mood, raising energy and reducing tension (out of 18 of the analysed strategies; [44]). Overall, many studies show music is recognized as an adaptive and effective mood regulator [10,11,45]. For example, previous research indicates that listening to music is a factor that contributes to altering depressed mood, reducing tension, reducing stress and anxiety, and it has been indicated that music can be a means for self-therapy [12,44,46,47,48]. Thus, music can be beneficial to a person’s health and well-being [49].

The above-mentioned differences between Cluster 1 and 3 seem to be related to what has been mentioned before-namely, people who reach for food under the influence of negative emotions most often choose comfort food-high-calorie food, unhealthy snacks and fast food [5,39]. This may be related to a sensation transfer effect [50] which is related to the fact that the emotional state experienced while listening to music also determines other behaviours (including food preferences; [51]). A similar effect could not be found for healthy eating behaviour. This is an interesting finding that may suggest that the emotional state may not be as strong a determinant of healthy food choices as we see with unhealthy choices. However, more research is needed in this area.

Referring to other (additional and not directly related to the hypothesis) results of our study, it can be indicated that participants with a high level of food-based regulatory strategies and a low level of music-based regulatory strategies (Cluster 3) had significantly greater levels of unhealthy eating behaviours, depression, anxiety and stress than those with a low level of food-based regulatory strategies and a low level of music-based regulatory strategies (Cluster 4). This may mean that this group (Cluster 4) used yet another (not included in the study) strategy of regulating the emotional state. However, to verify this in subsequent studies, the evaluation of the emotion regulation strategy should be extended to other methods (e.g., physical activity, alcohol, rumination, positive reappraisal etc.; [52]).

Interestingly, in addition, it turned out that participants with a high level of both of the above-mentioned strategies (Cluster 2) had a significantly higher level of depression, anxiety and stress than Cluster 1. One potential explanation may be that the positive effect related to the use of music is nullified by the interchangeable and/or equally frequent use of food as a regulator of the emotional state. However, this is only a supposition that needs to be verified in future studies.

It needs to be remembered that a food-based strategy of coping with negative emotions can result in negative health consequences, as supported by, among others, Psychosomatic Theory, which states that emotional eating provides only temporary separation from the negative emotions experienced [53]. A consequence of emotional eating can therefore be the onset of obesity [8], which can lead to other somatic illnesses.

Regarding the last two significant differences between the clusters, the results indicate that: (a) Cluster 4 had a higher level of unhealthy eating behaviours than Cluster 1; (b) Cluster 2 had a higher level of anxiety than Cluster 4. This means that, with regard to eating behaviour, people with a dominant tendency to regulate by music (Cluster 1) may function better than those who use other strategies (not included in this study; Cluster 4). Secondly, with regard to anxiety, people employing other strategies (than those included in the study; Cluster 4) may be more effective in reducing anxiety than those in whom the positive effect of using music can be offset by food-based strategy (Cluster 2). However, these are only potential explanations that require further analysis. particularly when we consider that disseminating information about the positive impact of music on the regulation of emotions and eating could be one of the supporting strategies in the field of protective mental health during a pandemic.

To sum up, the results obtained indicate that during the COVID-19 pandemic the group of people regulating their emotional state and unhealthy eating predominantly with food is potentially characterized by worse functioning than the group of people regulating with music. Therefore, it can be concluded that people who regulate their functioning using food should be included in preventive measures by specialists. During the visit, psychologists and primary care physicians can ask patients about their daily strategies and based on this information specialists can estimate the potential risk of developing high levels of stress and anxiety, depressive disorders and unhealthy eating habits and provide specific (match) intervention. Such information may therefore be important for planning prophylaxis and intervention programmes.

Regarding the limitations of the present study, future research could be expanded (in addition to the aspects already mentioned) through: (a) including information regarding the type of music listened to (i.e., because people who reported depression listened to negative music, which lowered their mood even more; [19]); (b) containing information on emotions other than only anxiety and stress (e.g., anger, happiness; [15]); (c) including in the analysis the age variable (i.e., because although gender was insignificant to the use of music as emotional regulation, age did matter; [10]) and body mass index (i.e., because BMI may be a factor that differentiates emotional functioning and eating habits; [54,55,56]); (d) planning longitudinal studies and/or experimental studies and/or ecological momentary assessment because such research may allow for a better understanding of cause and effect relations, as well as for a better analysis of the dynamic and trajectory of changes in emotional state and eating behaviours; (e) using also objective ways of measuring emotional state [57] and eating behaviours [58]. Finally, it should also be added that our results were developed based on data provided by volunteers, which also affects the possibility of generalizing these results. What is more, despite good reliability and validity of measures, it should be emphasized that the obtained results should be approached with caution, because almost all questionnaires have not been previously validated in a representative sample of the Polish population. The reference values of one population do not have to be valid for another population, therefore the results may have a significant bias.

## Figures and Tables

**Table 1:** Demographics characteristic.

	Cluster 1 (*N* = 148): + Low Food-Based + High Music-Based	Cluster 2 (*N* = 42): + High Food-Based + High Music-Based	Cluster 3 (*N* = 70): + High Food-Based + Low Music-Based	Cluster 4 (*N* = 150): + Low Food-Based + Low Music-Based		
	*M* (*SD*)				*F*	*Post hoc*
**Age**	35.86	28.79	28.23	30.22	F(3, 406) = 7.03,	1 vs. 2 * 1 vs. 3 *** 1 vs. 4 ** 2 vs. 3 2 vs. 4 3 vs. 4
	(14.53) ^a,b c^	(11.51) ^a^	(11.80) ^b^	(12.88) ^c^	*p* < 0.001	
**BMI**	24.07 (4.95)	23.71 (4.50)	24.87 (5.07)	23.70 (4.99)	*F*(3, 406) = 0.94, *p* > 0.05	1 vs. 2 1 vs. 3 1 vs. 4 2 vs. 3 2 vs. 4 3 vs. 4
	*N* (%)				*χ^2^* and Cramer’s *V*	
**BMI categories**					𝜒^2^_(9)_ = 13.88, Cramer’s *V* = 0.11 ^NS^	
Underweight	9 (6.08)	2 (4.76)	2 (2.86)	18 (12.00)		
Normal weight	87 (58.78)	29 (69.05)	37 (52.86)	83 (55.33)		
Overweight	38 (25.68)	7 (16.67)	20 (28.57)	28 (18.67)		
Obesity	14 (9.46)	4 (9.52)	11 (15.71)	21 (14.00)		
**Education**					𝜒^2^_(9)_ = 20.52, Cramer’s *V* = 0.13 *	
Elementary	0	0	1 (1.43)	0		
Vocational	1 (0.68)	0	0	3 (2.00)		
Secondary	42 (28.38)	16 (38.10)	33 (47.14)	69 (46.00)		
Higher	105 (70.94)	26 (61.90)	36 (51.43)	78 (52.00)		

If clusters share the same non-capital letter, then the differences between the groups are statistically significant. * *p* < 0.05, ** *p* < 0.01, *** *p* < 0.001; ^NS^ a non-significant.

**Table 2:** Separate univariate ANOVAs on the outcome variables.

	Cluster 1 (*N* = 148): + Low Food-Based (EOQ) + High Music-Based (B-MMR)	Cluster 2 (*N* = 42): + High Food-Based (EOQ) + High Music-Based (B-MMR)	Cluster 3 (*N* = 70): + High Food-Based (EOQ) + Low Music-Based (B-MMR)	Cluster 4 (*N* = 150): + Low Food-Based (EOQ) + Low Music-Based (B-MMR)		
	*M* (*SD*)				*F*	*Post hoc*
**HUEBS-HE**	50.91 (10.32)	49.98 (10.63)	53.30 (12.55)	53.90 (10.94)	*F*(3, 406) = 2.68, *p* > 0.05 *ƞ_p_^2^* = 0.02	1 vs. 2 1 vs. 3 1 vs. 4 2 vs. 3 2 vs. 4 3 vs. 4
**HUEBS-UE**	35.97 (11.37) ^a,b^	40.07 (11.31)	44.74 (12.84) ^a,c^	40.07 (11.65) ^b,c^	*F*(3, 406) = 9.27, *p* < 0.001 *ƞ_p_^2^* = 0.06	1 vs. 2 1 vs. 3 *** 1 vs. 4 * 2 vs. 3 2 vs. 4 3 vs. 4 *
**DASS-D**	9.55 (8.37) ^a,b^	15.00 (9.59) ^a^	17.54 (10.60) ^b,c^	11.39 (9.94) ^c^	*F*(3, 406) = 12.88, *p* < 0.001 *ƞ_p_^2^* = 0.09	1 vs. 2 ** 1 vs. 3 *** 1 vs. 4 2 vs. 3 2 vs. 4 3 vs. 4 ***
**DASS-A**	7.50 (7.00) ^a,b^	14.21 (7.45) ^a,c^	14.41 (8.87) ^b,d^	9.36 (7.95) ^c,d^	*F*(3, 406) = 17.21, *p* < 0.001 *ƞ_p_^2^* = 0.11	1 vs. 2 *** 1 vs. 3 *** 1 vs. 4 2 vs. 3 2 vs. 4 ** 3 vs. 4 ***
**DASS-S**	12.67 (8.97) ^a,b^	19.26 (8.73) ^a^	21.69 (10.91) ^b,c^	15.51 (9.50) ^c^	*F*(3, 406) = 16.21, *p* < 0.001 *ƞ_p_^2^* = 0.11	1 vs. 2 ** 1 vs. 3 *** 1 vs. 4 2 vs. 3 2 vs. 4 3 vs. 4 ***

B-MMR—the Brief Music in Mood Regulation Scale; EOQ—the Emotional Overeating Questionnaire; HUEBS—the Healthy and Unhealthy Eating Behavior Scale: health eating (HE), unhealthy eating (UE); DASS—the Depression, Anxiety and Stress Scale: depression (D), anxiety (A), stress (S). If clusters share the same non-capital letter, then the differences between the groups are statistically significant. * *p* < 0.05, ** *p* < 0.01, *** *p* < 0.001.

## Data Availability

The data that support the findings of this study are available from the corresponding author upon reasonable request.
